# Autologous Nanofat Indications in Wound Healing: A Systematic Review

**DOI:** 10.3390/biomedicines14061215

**Published:** 2026-05-28

**Authors:** Stefanie Bonini, Patricia Fuentes, Richard Brannon Claytor

**Affiliations:** 1 Claytor Noone Plastic Surgery, Bryn Mawr, PA 19010, USA; patty@cnplasticsurgery.com (P.F.); drclaytor@cnplasticsurgery.com (R.B.C.); 2 Herbert Wertheim College of Medicine, Florida International University, Miami, FL 33199, USA; 3 Plastic Surgery, Main Line Health, Bryn Mawr, PA 19010, USA

**Keywords:** autologous nanofat, wound healing, scar management

## Abstract

**Introduction**: Chronic wounds and pathologic scars remain a persistent challenge in plastic surgery. Conventional treatments can be costly and inconsistent, prompting interest in regenerative approaches that utilize autologous tissue. Emulsified fat produces nanofat through mechanical processing and contains adipose-derived stem cells, stromal vascular fractions, extracellular matrix proteins, cytokines and growth factors. The purpose of this systematic review is to evaluate the use of autologous nanofat for wound healing and scar management, with emphasis on preparation techniques, treatment indications, and outcomes. **Methods**: A comprehensive PubMed search with no date restrictions was conducted in January 2026 using MeSH terms and keywords related to nanofat and wound-healing applications. Studies were screened independently by two reviewers using the Rayyan platform. Eligible studies evaluated nanofat for wound healing in human or animal subjects; non-English articles, studies not involving nanofat, editorials, and conference abstracts were excluded. The extracted data included study characteristics, participant numbers, treatment details, indications, adjunct therapies, follow-up duration, outcomes, and complications. Studies were grouped by clinical application, with individual reports included in multiple categories when relevant. **Results**: The search identified 53 records, of which 22 studies met the inclusion criteria after screening. These included 20 human and two animal studies spanning randomized controlled trials (n = 3), prospective trials (n = 6), retrospective analyses (n = 6), case series (n = 4), and case reports (n = 3). Mechanical emulsification was the predominant autologous nanofat preparation method (91%), often combined with filtration or centrifugation. Clinical indications in human studies were diverse, most commonly including scar treatment (n = 14) (acne, burns, depressed, and post-surgical), followed by chronic wounds (n = 3) and reconstructive applications (n = 3). Nanofat was administered via injection in 86% of studies (n = 19), typically using fine-gauge needles or microcannulas with intradermal or subdermal placement, while three studies used non-injection approaches such as topical, membrane, or dressing-based delivery. Scar or aesthetic parameters, measured using VSS, POSAS, physician grading, photography, pigmentation analysis, or clinical appearance, were evaluated in 73% of studies (n = 16), and all reported improvement in variables such as pigmentation, pliability, thickness, texture, or overall appearance. Wound-healing endpoints were assessed in 36% (n = 8), with 100% (n = 8) demonstrating accelerated healing, improved epithelialization, or defect closure. Patient-reported outcomes, including satisfaction or quality of life, were measured in 32% (n = 7), and all showed improvement. Objective imaging modalities (e.g., 3D imaging, ultrasound, angiography, digital analysis) were used in 23% (n = 5), each confirming structural or physiologic improvement. Histologic or biomolecular analyses were performed in 27% (n = 6) and uniformly demonstrated regenerative changes, such as increased angiogenesis, collagen remodeling, or growth factor expression. Treatment was well tolerated, with 77% of studies (n = 17) reporting minimal or no complications and only transient mild adverse effects, including mild pain, bruising, erythema, and edema. **Conclusions**: Current evidence suggests that autologous nanofat is a promising regenerative therapy for wound healing and scar modulation. Across diverse clinical applications, nanofat has been associated with improved tissue quality, enhanced healing, and favorable patient-reported outcomes, with minimal complications. The mechanical processing of autologous tissue may also involve fewer regulatory concerns compared with more extensively manipulated cellular products.

## 1. Introduction

Chronic wounds and scars remain a significant clinical challenge in plastic surgery, often resulting from impaired healing due to ischemia, inflammation, or infection. At the same time, current scar management strategies are frequently associated with delayed healing, variable aesthetic outcomes, and high treatment costs. These limitations have driven growing interest in regenerative medicine approaches aimed at enhancing tissue repair while improving cosmetic outcomes [1,2].

Mechanical processing of lipoaspirate, originally described by Tonnard et al. [3], has led to the development of autologous nanofat, an emulsified adipose derivative with reduced particle size that allows for intradermal delivery while preserving regenerative cellular components [2,3,4]. This technique involves mechanical emulsification of harvested adipose tissue and is considered minimal manipulation of lipoaspirate under the U.S. Food and Drug Administration [5]. Adipose tissue contains a heterogeneous stromal vascular fraction (SVF) composed of adipose-derived stem cells, endothelial progenitor cells, pericytes, cytokines, and growth factors. These biologic components have been shown to promote angiogenesis, regulate inflammatory signaling, and stimulate extracellular matrix remodeling, processes that are essential for effective wound healing and tissue regeneration [6,7,8,9]. Through these mechanisms, nanofat may have the potential to enhance wound repair by improving vascularization, modulating inflammation, and stimulating dermal remodeling in damaged tissue. By enriching the wound environment with regenerative cells and bioactive signaling molecules, nanofat may facilitate activation of the wound-healing cascade and improve tissue regeneration.

Nanofat therapy has therefore been investigated across a broad spectrum of clinical indications, including acne scars, post-burn scars, surgical scars, chronic ulcers, and aesthetic skin rejuvenation [10,11,12,13,14]. Early clinical studies report improvements in scar quality, accelerated wound healing, and increased patient satisfaction following treatment [15,16,17]. Experimental and histologic studies further support these findings, demonstrating enhanced angiogenesis, epithelial regeneration, and extracellular matrix remodeling after nanofat administration [8,18].

The purpose of this systematic review is to evaluate the use of autologous nanofat for wound healing and scar management, with emphasis on preparation techniques, treatment indications, and reported outcomes.

## 2. Materials and Methods

### 2.1. Data Search

A comprehensive literature search was performed using PubMed (National Library of Medicine, Bethesda, MD, USA; https://pubmed.ncbi.nlm.nih.gov/, accessed 20 January 2026). No date restrictions were applied. The search was completed in January 2026 using a combination of MeSH terms and keywords related to nanofat and application techniques. The complete search strategy included: ((“nanofat” OR “autologous nanofat”) AND (“wound healing” OR “wound repair” OR “tissue regeneration” OR “soft tissue regeneration” OR “scar”) AND (human OR humans)). This review was registered on PROSPERO (CRD420261336183).

### 2.2. Study Selection

Articles were screened using the Rayyan platform (Rayyan Systems Inc., Cambridge, MA, USA) by two independent reviewers. Studies were eligible for inclusion if: (1) evaluated nanofat on wound healing and (2) studies including human or animal participants. Exclusion criteria included non-English language publications, studies that did not mention nanofat, editorials, and conference abstracts.

### 2.3. Risk Assessment

Risk of bias was independently assessed according to study design using criteria adapted from the Newcastle–Ottawa Scale for observational studies, the Cochrane Risk of Bias framework for randomized trials, and JBI critical appraisal tools for case reports and case series. Studies were qualitatively evaluated based on methodological characteristics, including sample size, presence of control groups, randomization, blinding, follow-up adequacy, outcome assessment, and reporting transparency (Supplementary Table S1) [19,20,21].

### 2.4. Data Extraction

From each included study, the following data were extracted: reference, country of publication, number of participants, amount of nanofat applied, wound type, indication, presence of any adjunctive treatments, follow-up time, main findings, and complications. Studies were categorized by application, and individual studies were included in multiple categories when they addressed more than one clinical application. This review was conducted according to PRISMA guidelines.

## 3. Results

### 3.1. Search Results

**Figure 1:**
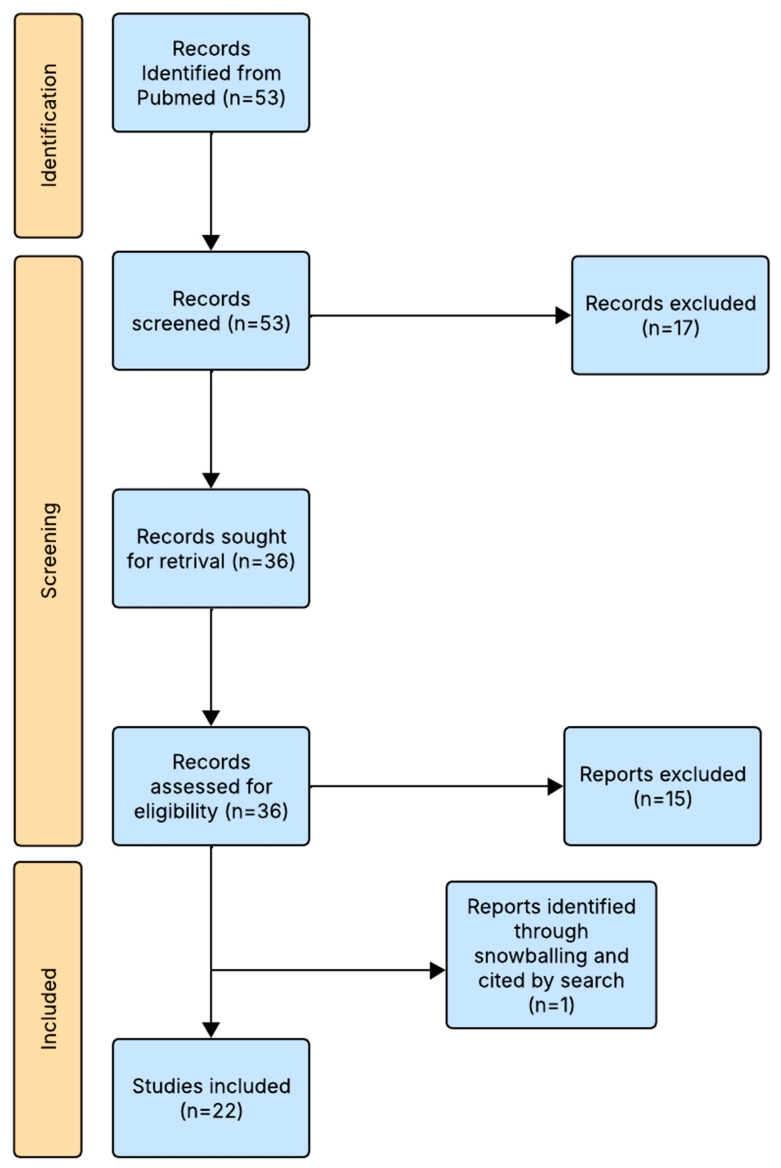
Flow chart of the systematic literature search according to PRISMA guidelines. A total of 53 records were identified through PubMed and were then screened by title and abstract. Of these, 36 were selected for full-text review. One study was identified through the snowballing method. Ultimately, 22 studies met the inclusion criteria and were included.

The initial search identified 53 publications using PubMed. These publications were screened based on their titles and abstracts. The selection was based on relevance to nanofat and its applications. A total of 36 publications were included for full text screening, and 21 studies met inclusion criteria (Figure 1 PRISMA flow diagram). One additional study was identified through a snowball search and was incorporated into the final analysis for a total of 22 studies (Table 1).

### 3.2. Study Characteristics

A total of 22 studies were included in this review, comprising 20 human studies and two animal studies. The study design included randomized controlled trials (n = 3), prospective trials (n = 6), retrospective analyses (n = 6), case series (n = 4), and case reports (n = 3). The follow-up duration varied across studies (1 day to 1 year) depending on indication and study type.

### 3.3. Nanofat Preparation Techniques

Mechanical emulsification was used in 91% (n = 20) of studies. This process typically involved transferring harvested lipoaspirate between two syringes approximately 30 times through Luer-lock connectors or progressively smaller adapters to produce a homogenized injectable suspension [10,12,27]. Several authors specifically described using the Tonnard technique, a standardized emulsification protocol designed to generate nanofat [2,4,18].

Filtration following emulsification was frequently performed using fine mesh filters to obtain a uniform liquid product suitable for intradermal injection [10,11,13]. Some protocols incorporated centrifugation either before or after emulsification to remove oil and aqueous fractions or to further refine the graft [9,25,29]. One experimental animal study used mechanical blade fragmentation of adipose tissue prior to emulsification [8], whereas a clinical case report omitted filtration entirely [26].

Nanofat was administered via injection in 86% of studies (n = 19), typically using fine-gauge needles or microcannulas with intradermal or subdermal placement, while three studies used non-injection approaches such as topical, membrane, or dressing-based delivery [13,18,28].

### 3.4. Risk Bias

Most included studies were determined to have moderate-to-high risk of bias. Of the 22 included studies, nine were categorized as high risk, 11 as moderate risk, and only one randomized controlled trial was considered low risk of bias. Common limitations included retrospective study design, small sample sizes, lack of comparator groups, subjective outcome assessment, limited blinding, and heterogeneous treatment protocols. Several studies additionally incorporated adjunctive therapies, making it difficult to isolate the independent effects of nanofat. Animal studies were considered limited in external validity due to challenges translating findings to human wound healing.

### 3.5. Human Studies

#### Wound Types and Clinical Indications 

Clinical indications were diverse, and most commonly included scar treatment (n = 14), followed by chronic wounds (n = 3) and reconstructive applications (n = 3) . Scar treatment was the most common application and included acne scars, burn scars, depressed scars, and mixed etiologies [9,10,12,23,29]. Post-surgical scars were evaluated in studies of breast reduction scarring and soft palate fibrosis [17,26]. Chronic wounds such as hypertensive ischemic ulcers and diabetic ulcers were also treated [2,13]. One clinical study evaluated nanofat application in burn injuries [1]. Reconstructive indications extended to correction of lip deformities and treatment of thromboangiitis obliterans in a 48-year-old man [14,25].

### 3.6. Variables Measured

Outcomes assessed included clinical, patient-reported, imaging, and histology. Scars were evaluated in 73% of studies (n = 16), using validated instruments such as the Vancouver Scar Scale (VSS) and the Patient and Observer Scar Assessment Scale (POSAS), along with physician grading and photo comparisons [2,4,9,11,15,16,17,23,29]. Wound-healing endpoints were assessed in 36% of studies (n = 8), including time to closure, epithelialization, granulation tissue formation, and defect size reduction.

Patient-reported outcomes, including satisfaction and quality-of-life measures, were evaluated in 32% of studies (n = 7) using satisfaction questionnaires, Likert scales, FACE-Q, or SCAR-Q instruments [2,10,12,13,16,22,27]. Objective imaging modalities were used in 23% of studies (n = 5), each confirming structural or physiologic improvement. These included three-dimensional imaging to quantify scar concavity [23], Antera imaging to measure indentation index and erythema [11], ImageJ software for pigmentation analysis [15], ultrasound assessment of dermal thickness [24], and CT angiography [14]. Histologic or biomolecular analyses were performed in 27% of studies (n = 6).

### 3.7. Scar Management

Across studies assessing scar-related outcomes, all reported improvement in pigmentation, pliability, thickness, texture, or overall appearance. Significant VSS reductions were documented, including improvements in pliability (*p* = 0.006), height (*p* < 0.001), and total score (*p* < 0.001) [9]. In a case report of severe acne scarring, the patient’s VSS score decreased from 5.67 to 4.0 at one year post-treatment [4]. In a single-center prospective study of patients undergoing breast reduction, scars were treated immediately postoperatively and patients were divided into three groups: control (no treatment), fat graft injection alone, and fat graft enriched with nanofat. Comparative analysis demonstrated significantly lower Vancouver Scar Scale (VSS) scores in the nanofat-enriched group compared with controls. Additionally, pigmentation scores improved significantly in the nanofat-enriched fat graft-treated group (*p* = 0.005) [17].

The POSAS, used in four studies, provided both patient and observer reported evaluations of scar quality, with lower scores reflecting closer resemblance to normal skin [15,16,23,29]. POSAS scores similarly improved across studies. Scores decreased from approximately 6.8 to 2.6 at 12 months in post-burn alopecia cases (*p* < 0.00001) [16], and from 28.80 to 12.20 for patients and from 18.00 to 9.20 for observers in atrophic facial scars (*p* ≤ 0.001) [29]. Significant improvements in pliability and pigmentation were also observed in burn scars (*p* < 0.0001) [15]. At three months following nanofat injection for depressed scars, physician assessments demonstrated complete healing in 30% of cases, marked improvement in 41%, and moderate improvement in 20% of patients [12]. In a similar study assessing facial and body scars following nanofat injection, independent reviewers rated 74% of treated scars as good and 18% as satisfactory [27].

Pain intensity was assessed in two studies using the Visual Analog Scale (VAS), enabling quantitative monitoring of symptom change over time [2,17]. In a reconstructive aesthetic protocol combining CO_2_ laser resurfacing, microneedling, and nanofat (LaMiNa), patients demonstrated improvement in fine lines and rhytids with rapid recovery free of pain, and histologic analysis revealed faster epithelial recovery and reduced dermal inflammation compared with laser treatment alone [28].

In a single-center retrospective study of depressed facial scars, Qu et al. (2025) [23] utilized three-dimensional imaging and a computerized skin analysis system to objectively quantify scar concavity, elasticity, and collagen characteristics, demonstrating significant improvement at six months post-treatment. Similarly, in a prospective study of post-burn scars, Rageh et al. (2025) [11] employed Antera 3D imaging to measure indentation index and erythema, documenting significant reductions in both parameters (*p* < 0.001). In a randomized controlled clinical trial evaluating nanofat-treated acne scars, Behrangi et al. [24] used high-frequency ultrasound to assess epidermal, dermal, and total skin thickness, reporting significantly greater dermal and total thickness in treated groups at one month (*p* = 0.042 and *p* = 0.040).

### 3.8. Wound-Healing Management

Six studies evaluating wound healing reported improvement [1,2,13,14,26,28]. In hypertensive ischemic ulcers, healing, as measured by mean percentage reduction in ulcer surface area, reached 89.29% at 3 months and 99.29% at 6 months (*p* < 0.0001), with associated pain reduction [2]. In acute burn patients, nanofat reduced contracture formation and improved scar texture and pigmentation [1].

Reconstructive and functional applications (n = 4 clinical reconstructive studies) also demonstrated favorable outcomes. In upper lip reconstruction following hemangioma treatment, nanofat combined with condensed fat grafting improved lip symmetry, contour, and smoothness on long-term follow-up [25]. In a reconstructive ischemic limb case, Benjamin Ng et al. [14] performed three-dimensional computed tomography angiography before and after nanofat application. Results demonstrated restoration of radial artery patency alongside clinical wound improvement. In velopharyngeal incompetence secondary to scarring, autologous nanofat injection achieved complete velopharyngeal closure within three months, with normalization of speech parameters maintained at follow-up [26]. Regenerative nanofat membrane application in complex reconstructive wounds, including cleft palate and skin necrosis, was associated with accelerated healing and high patient satisfaction [13].

### 3.9. Histology

In a prospective case series of atrophic facial scars, Gu et al. [29] immunohistochemical analysis, revealed increased basal layer melanin density (*p* = 0.01), collagen remodeling, and postoperative appearance of sebaceous and sweat glands absent preoperatively. Additional studies found increased collagen deposition and elastic fiber remodeling [1,9], following the application of nanofat on scars or dermal burns.

### 3.10. Complications

Treatment was well tolerated, with 77% of studies (n = 17) reporting minimal or no complications. Mild pain was reported following nanofat injection in two studies when patients were asked to rate pain as mild, moderate or severe [10,11]. Transient bruising, erythema, or edema occurred in four studies but resolved spontaneously without sequelae [9,10,12,15]. One study reported hematoma formation in two patients after nanofat application, however this study consisted of an acupotomy subcision before nanofat was applied [23]. No adverse events or long-term complications were documented across included studies.

### 3.11. Animal Studies

#### Wound Healing

Two additional studies were experimental animal studies evaluating burn and full-thickness wound healing [8,18]. In an experimental full-thickness wound model in rabbits, Hidayati et al. [18] measured epidermal growth factor levels using enzyme-linked immunosorbent assay and demonstrated significantly elevated EGF concentrations on days 3 and 7 (*p* < 0.001) in nanofat-treated wounds.

In a randomized controlled animal study using a murine third-degree burn model, Ivari and Mahdipour [8] evaluated the regenerative effects of nanofat compared with stem cell–derived small extracellular vesicles and untreated controls. Animal burn models demonstrated significantly greater wound closure by day 21, enhanced granulation tissue formation, increased epithelial thickness, and approximately threefold higher CD34-positive vessel density in nanofat-treated wounds compared with controls (Section 3.5, Section 3.6, Section 3.7, Section 3.8, Section 3.9, Section 3.10 and Section 3.11 are summarized in Table 2).

## 4. Discussion

This review suggests that autologous nanofat therapy may improve wound healing and scar modulation across a wide range of wound types, scar etiologies, and reconstructive indications. Several studies reported sustained clinical improvement during follow-up, suggesting potential regenerative effects beyond temporary cosmetic improvement. Collectively, these findings support the possibility that nanofat may function as a regenerative biologic therapy capable of enhancing tissue remodeling and wound healing. However, the included studies varied considerably in design, patient population, wound type, and outcome assessment, and therefore the findings should be interpreted with caution.

Among all reported outcomes, improvements in scar quality were the most consistent and reproducible findings. Improvements in pigmentation, pliability, thickness, and overall appearance were demonstrated using validated instruments such as the VSS and POSAS [9,11,16,29]. Several studies also supported these findings with objective measurements. Imaging analyses demonstrated reductions in scar concavity and erythema, while ultrasound studies showed increased dermal thickness following treatment [11,23,24]. Histologic studies additionally reported collagen remodeling, elastic fiber reorganization, and regeneration of dermal structures following nanofat application [9,29]. Kemaloğlu et al. [17] compared untreated scars, fat grafting alone, and nanofat-enriched fat grafting following breast reduction surgery and reported superior scar outcomes in the nanofat-enriched group, suggesting that nanofat may provide additional regenerative benefits when combined with fat grafting.

The proposed regenerative effects of nanofat are supported by its biologic composition. Tonnard et al. [3] demonstrated that although mechanical emulsification disrupts mature adipocytes, it preserves stromal vascular fraction (SVF) components, including adipose-derived stem cells (ASCs), endothelial progenitor cells, and pericytes. These cellular components are believed to contribute to tissue repair through secretion of cytokines and growth factors involved in angiogenesis, inflammation regulation, and extracellular matrix remodeling [6,30]. Adipose-derived stem cells also appear to have immunomodulatory effects that may reduce excessive inflammatory signaling and support a reparative wound-healing environment [6,30]. These mechanisms may help explain the improvements in scar appearance and tissue quality reported in clinical studies.

Animal studies provided additional experimental support for these regenerative mechanisms. Experimental wound models demonstrated increased CD34-positive vessel density, elevated epidermal growth factor (EGF) levels, improved epithelial thickness, and accelerated wound closure following nanofat treatment [8,18]. These findings suggest that nanofat may enhance angiogenesis and epithelial regeneration during wound repair. However, because these studies were performed in animal models, their findings may not fully reflect the complexity of human wound healing and should therefore be interpreted cautiously.

Adequate vascularization is essential for wound healing because newly formed blood vessels provide oxygen, nutrients, immune cells, and regenerative signaling molecules to injured tissue. Therapies that improve angiogenesis may therefore be particularly useful in ischemic or poorly vascularized wounds [31]. By delivering stem cells, cytokines, and growth factors directly into damaged tissue, nanofat may help support neovascularization and activation of the wound-healing cascade. This may partially explain the favorable outcomes reported in studies involving chronic ulcers, ischemic wounds, and burn injuries.

Additional support for a physiologic mechanism comes from studies reporting reductions in pain and restoration of functional capacity following nanofat treatment. Improvements in symptoms and functional outcomes, including decreased pain scores, suggest that nanofat may influence vascular and inflammatory processes within treated tissues [2,14,28]. Pain in wounds is strongly influenced by inflammation and ischemia, both of which can sensitize peripheral nerves and increase nociceptor activation [32]. Claytor et al. [28] found that introducing autologous nanofat into the reticular dermis immediately after CO_2_ laser treatment and microneedling resulted in a virtually pain-free clinical experience for patients. This suggests autologous nanofat may contain mediators which ameliorate nociceptor pain response. The reductions in pain reported across multiple studies suggest that nanofat may alleviate wound associated pain by promoting angiogenesis and limiting cytokine-driven nociceptor sensitization.

In addition to its regenerative potential, nanofat may provide some practical economic advantages because it relies on autologous adipose tissue processed through relatively simple mechanical techniques. Although nanofat is not an inexpensive therapy due to the need for liposuction, processing, and procedural expertise, it may offer longer-lasting regenerative effects compared with standard wound care therapies, which often require repeated applications or prolonged treatment courses. Additionally, because Nanofat is harvested directly from the patient, large amounts of regenerative material can be obtained during a single procedure without dependence on manufactured biologic products. Compared with advanced therapies such as negative pressure wound therapy (NPWT) and hyperbaric oxygen therapy, which often require multiple treatment sessions and specialized equipment, nanofat may represent a more durable regenerative option despite higher upfront procedural costs. This consideration is particularly relevant given the growing financial burden associated with chronic wound care [33,34,35].

Nanofat therapy demonstrated a favorable safety profile across studies, with most reporting no complications or only mild effects, such as localized pain, bruising, erythema, or edema. Commercially available wound-healing products include advanced dressings and topical agents. These products focus on maintaining a moist environment and reducing infection; however, they do not directly target the underlying cellular mechanisms that drive tissue regeneration and wound healing. Other products, such as enzymatically isolated SVF, involve more extensive manipulation and are subject to stricter regulatory oversight due to concerns regarding contamination, when compared to mechanical isolation [5,30]. Given its autologous origin and the low rate of reported complications, nanofat appears to have a favorable safety profile among currently available regenerative therapies.

### Limitations and Future Studies

Substantial heterogeneity existed among the included studies, particularly with respect to nanofat preparation protocols, adjunctive therapies, injection techniques, wound types, outcome measures, patient populations, and duration of follow-up (Table 3). Due to heterogeneity, quantitative meta-analysis was not feasible. This variability limited direct comparison between studies and reduced the ability to draw standardized conclusions regarding the efficacy of nanofat for wound healing.

Several studies combined nanofat with adjunctive biologic therapies, including platelet-rich fibrin and stromal vascular fraction concentrates [13,22]. In other studies, nanofat was used in combination with surgical or procedural interventions, such as microneedling, laser resurfacing, fat grafting, and reconstructive procedures, to augment tissue regeneration and aesthetic outcomes [4,24,25,28]. While these adjunctive approaches often yielded strong outcomes, they make it difficult to determine the independent therapeutic contribution of nanofat itself. The frequent use of nanofat in combination with other biologic compounds or procedural interventions represents a limitation of the current literature and restricts the ability of this review to fully isolate the effects of nanofat on wound healing.

The search strategy was limited to the PubMed database, which may have introduced selection bias and restricted the comprehensiveness of the review. As a result, relevant studies indexed in other databases, such as Embase, Scopus, Web of Science, and Cochrane Library, may have been missed. This limitation should be acknowledged, as it reduces the likelihood that all pertinent literature on nanofat and wound healing was captured, and may impact the completeness and generalizability of the findings.

Most included studies were observational in design and involved small sample sizes. Outcome measures varied widely across studies, and preparation protocols differed. This variability limited direct comparison between studies. The findings of this review should be interpreted in light of the methodological limitations of the included studies. Many studies were case reports or case series with small cohorts and lacked control groups or blinding, increasing the risk of selection and measurement bias.

Future studies with larger randomized controlled trials are needed to understand the effect of nanofat on wound healing and scar management. Comparative studies evaluating nanofat against microfat, isolated stromal vascular fraction, or platelet-based therapies would be of interest. Other investigations could further define optimal treatment parameters and determine the specific cytokine and angiogenic pathways involved.

## 5. Conclusions

Current evidence suggests that autologous nanofat is a promising regenerative therapy for wound healing and scar modulation. Across multiple clinical contexts, nanofat has been shown to improve tissue quality, accelerate healing, and achieve high levels of patient satisfaction, with a strong safety profile.

## Figures and Tables

**Table 1:** Study type, indications and application techniques.

Title	Author	Year	Study Type	# Patients	Human/Animal	Adjunctive Treatments/Procedures	Wound Type	Application Technique
Role of Autologous Nanofat Grafting in the Treatment of Post Acne and Post Burn Scarring of the Face	Khan et al. [10]	2024	Case series	24	Human	none	post-acne and post-burn facial scars	Intradermal nanofat injections using 25 G injecting cannula
Evaluating the Efficacy of facial scar Treatment Techniques using Nanofat Grafting: A Case Series	Alnemr et al. [22]	2024	Case series	4	Human	platelet-rich fibrin	facial scars	Injecting unfiltered nanofat combined with platelet-rich fibrin (PRF) into the scar tissue.
Effectiveness of Acupotomy Subcision and Nanofat Grafting in Depressed Scars Treatment	Qu et al. [23]	2025	Single-Center retrospective analysis	32	Human	none	depressed facial scars	A 27-gauge, 10 mm needle and fat injection gun were employed, utilizing a retrograde linear threading technique applied parallel to the skin within the space created by needle knife. Each trigger administered approximately between 0.05 and 0.1 mL of the nanofat. Injections were administered directly into the subcutaneous space created by the acupotomy subcision.
Is Nanofat the Long-Awaited Treatment for Hypertensive Ischemic Leg Ulcers?	Moris et al. [2]	2025	Single-Center retrospective pilot study	23	Human	none	hypertensive ischemic leg ulcer	Using a 1-mL syringe and a 30-gauge needle the nanofat was injected intradermally on the edges around the ulcer and under the wound bed at a rate of 0.1 mL every 2 cm.
Can a Combination of Nanofat and Freeze-Dried Human Amniotic Membrane Enhance Full-thickness Wound Healing? An Animal Study Using rabbit Models	Hidayati et al. [18]	2024	Post-test control experimental study	36	Animal	freeze-dried human amniotic membrane (FDHAM)	full-thickness wound on back	Injected directly into the wound bed using 1 mL syringes, with 0.2 mL administered per wound to ensure even distribution.
Regenerative Nanofat Membrane Development Process	Fakih-Gomez et al. [13]	2025	Retrospective analysis	172	Human	nanofat combined with PRF	diabetic foot ulcers, cleft palate surgeries, facial dermabrasion, skin necrosis, revision rhinoplasties, and post-cosmetic surgery complications.	Membrane directly placed and sutured over the desired area.
Efficacy and Safety of Autologous Nanofat Injection in the Treatment of Postburn Scars Using Optical Skin Imaging Analysis	Rageh et al. [11]	2025	Prospective trial	30	Human	none	post-burn scars	Nanofat was injected into the superficial intradermal (using 28 G needles) and subdermal (using 23 G cannula) layers.
Camouflage of Postburn Scarring Alopecia Using Nanofat Grafting and Follicular Unit Hair Transplantation	Evin et al. [16]	2024	Patient series	18	Human	none	postburn scarring alopecia in and around the beard	27-gauge sharp needles that released and broke down the scar, creating a new space, into which the nanofat was injected until the appearance of yellowish discoloration of the scarred skin.
The Investigation of the Efficacy and Safety of Stromal Vascular Fraction in the Treatment of Nanofat-Treated Acne scar: A Randomized Blinded Controlled Clinical Trial	Behrangi et al. [24]	2022	Randomized, single-blinded clinical trial	7	Human	SVF	acne facial scars	The scars were treated on one side of the face with nanofat subcutaneously and on the other side of the face with a combination of nanofat subcutaneously and SVF intradermally.
Nanofat and Platelet-Rich Plasma Injections Used in a Case of Severe Acne Scars	Pons et al. [4]	2022	Case report	1	Human	PRP/nanofat combination	acne scars	The injections were realized in the dermis using micro cannulas (after needle insertion). The temporal areas were priorly injected (due to greater atrophy in these areas) with 8 mL each and the glabella with 3 mL.
Effect of Autologous Fat Transfer in Acute Burn Wound Management: A Randomized Controlled Study	Abouzaid et al. [1]	2021	Prospective, open-label single center, randomized control clinical trial	100	Human	none	deep dermal burns	Single injection of autologous fat grafting, and the wounds were then dressed with nanofat.
Application of Nanofat Grafting to Rescue a Severe Ischaemic Hand with Thromboangiitis Obliterans	Benjamin Ng et al. [14]	2021	Case report	1	Human	fasciotomy and necrotic tissue debridement	cyanosis in both hands and the right foot	A 16 G blunt cannula was used to administer the fat while withdrawing MAFT-GUN. Each delivered fat parcel was set at 1/60 mL (each parcel volume, 0.017 mL) and was dispersed throughout his left forearm from the proximal to distal end along both the radial and ulnar sides through intramuscular injection.
Autologous Nanofat Injection in Treatment of Scars: A Clinicohistopathological Study	Rageh et al. [9]	2021	Prospective trial	30	Human	none	traumatic origin scars (63%), post-burn scars (10%), post-acne scars (23%), and post-chicken pox scars (3.3%)	Superficial intradermal nanofat injection was done by 1-mL (28 G) insulin syringes, in addition to subdermal nanofat injection using (21 G) cannula mounted on a Luer-Lock syringe.
Adipose Tissue Versus Stem Cell-Derived Small Extracellular Vesicles to Enhance the Healing of Acute Burns	R. Ivari, Mahdipour [8]	2021	Randomized control trial	54	Animal	none	mouse model third degree burn	200 μL of the prepared NF tissue (~250 mg of fat) was injected subcutaneously at the burn site of each mouse in the related experimental group.
Upper Lip Reconstruction Utilizing a Two-Stage Approach with Nanofat Grafting After Hemangioma Treatment	Qi et al. [25]	2021	Retrospective study	24	Human	flap transfer	lip deformities	A 1 mL syringe fitted with a 1.5 mm blunt syringe needle was used to implant the nanofat through a pinhole located at the ipsilateral corner of the mouth following a multiplate and multitunnel microinjection technique.
Nanofat Injection for the Treatment of Depressed Facial Scars	Huang et al. [12]	2021	Retrospective study	52	Human	none	depressed facial scars	Injected nanofat graft material into the scar with a 27-gauge needle and a 1-mL syringe in a fanwise pattern to increase the thickness of the dermis.
Immediate Fat and Nanofat-Enriched Fat Grafting in Breast Reduction for Scar Management	Kemaloğlu et al. [17]	2021	Single center prospective study	45	Human	none	breast reduction surgical scars	Fat grafts injected under surgical incisions.
Adding Nanofat to Fat Grafting to Treat Velar Scarring in Velopharyngeal Incompetence	Cantarella et al. [26]	2020	Case report	1	Human	microfat	velar scars	A total of 0.7 to 1 mL of nanofat is inserted within the scar tissue.
Unfiltered Nanofat Injections Rejuvenate Postburn Scars of Face	Jan et al. [15]	2018	Prospective study	48	Human	none	post-burn facial scars	Pretunneling (subcision) was done in the intradermal or subdermal layer depending on the thickness of the dermis. Fat was injected in a fanwise pattern with an 18-gauge needle connected to a 1-mL syringe until the skin blanched or displayed a yellowish discoloration.
Nanofat Grafting for Scar Treatment and Skin Quality Improvement	Uyulmaz et al. [27]	2018	Single center retrospective study	52	Human	none	facial and body scars	The obtained liquid was injected with 24, 25, or 27-gauge sharp needles into the scar tissue or the dermis.
LaMiNa: A Creative Synergistic Approach to Facial Rejuvenation	Claytor et al. [28]	2023	Single center prospective study	23	Human	CO_2_ lasering followed by microneedling	perioral rhytids	Nanofat was applied to the skin and the microneedling procedure repeated to allow penetration through the epidermis. Repeat applications of nanofat were applied after the prior aliquot had been absorbed into the tissue for a total of 20 cc of autologous nanofat.
Use of Condensed Nanofat Combined with Fat Grafts to Treat Atrophic Scars	Gu et al. [29]	2018	Prospective case series	20	Human	none	atrophic facial scars	Injected condensed nanofat grafts intradermally into scars using 29-gauge insulin syringes (BD [Becton, Dickinson, and Company]) to increase the thickness of tissue under the epidermis and dressed condensed nanofat on the surface of incisions.

**Table 2:** Study follow-up duration, variables and results.

Title	Author	Follow-Up Time	Variables Measured	Main Results	Complications
Role of Autologous Nanofat Grafting in the Treatment of Post Acne and Post Burn Scarring of the Face	Khan et al. [10]	3 months, 6 months	scar hardness/thickness, hyperpigmentation, softness (determined by surgeon) and patient satisfaction (yes/no)	Acne scars softer and less visible (61.5%), reduction of hyperpigmentation (38.4%). Burn scars softer (54.5%), reduced hyperpigmentation (36.3%). 75% of patients were highly satisfied.	mild pain (33.3%), bruising (20.8%), and oozing from the wound (8.3%)
Evaluating the Efficacy of facial scar Treatment Techniques Using Nanofat Grafting: A Case Series	Alnemr et al. [22]	6 months	scar appearance	The injection of unfiltered nanofat combined with PRF into atrophic facial scars produced better cosmetic outcomes and decreased the defective appearance. After the follow-up, patients were extremely satisfied with both approaches.	none
Effectiveness of Acupotomy Subcision and Nanofat Grafting in Depressed Scars Treatment	Qu et al. [23]	6 months	measurement of scar concavity using a 3D camera, Patient Observer Scar Assessment Scale (POSAS), and collagen/elastin levels measured by a CBS skin analysis system	Scar concavity before treatment was significantly smaller than that of the control group (*p* = 0.021). Total POSAS scores differed significantly in terms of baseline and 6 months post-operation (*p* < 0.05). There was no difference in patient scores for color and thickness or in observer scores for pigmentation. Differences were observed in the mean values of skin collagen and elasticity at 6 months post-treatment compared to pre-treatment (*p* < 0.05).	two cases of hematoma (6.25%)
Is Nanofat the Long-Awaited Treatment for Hypertensive Ischemic Leg Ulcers?	Moris et al. [2]	6 months	wound-healing rate, pain reduction, Visual analog Scale (VAS), SCARQ and the Vancouver Scar Scale	The average wound-healing rate was 89.29% at 3 months, improving to 99.29% at 6 months (*p* < 0.0001). The mean VAS pain scores significantly decreased from an initial score of 5.87 to 0.39 at 3 months post-injection (*p* < 0.0001). Quality of life was significantly improved after treatment, as underscored by higher SCAR-Q scores and lower Vancouver scale scores.	none
Can a Combination of Nanofat and Freeze-Dried Human Amniotic Membrane Enhance Full-Thickness Wound Healing? An Animal Study Using Rabbit Models	Hidayati et al. [18]	days 3, 7 post-injury	epithelialization rate and measurement of epidermal growth factor (EGF) levels in the wounds	Combination (nanofat + FDHAM) treatment significantly elevated EGF levels in the wounds on both days 3 and 7 (all *p* < 0.001). No differences in epithelization.	none
Regenerative Nanofat Membrane Development Process	Fakih-Gomez et al. [13]	1 to 16 months	time to complete healing and patient satisfaction	Patient satisfaction was high. For skin necrosis, wound healing occurred within an average of 3 weeks. In cleft palate cases, complete closure within 2 weeks. For ear skin necrosis, the final healing process took 2 months. In diabetic foot patients, healing times ranged from 1 to 3 months for smaller defects and up to 6 months for larger defects.	none
Efficacy and Safety of Autologous Nanofat Injection in the Treatment of Postburn Scars Using Optical Skin Imaging Analysis	Rageh et al. [11]	4 months	skin’s overall roughness, as well as the depth and severity of scars, indentations, wrinkles, pigmentation, and redness (via 3D imaging) and the Vancouver Scar Scale (VSS)	There was a significant difference in the VSS before and after treatment, with a mean of 9.4 before and 6.1 after (*p* < 0.001). A significant improvement was noted in both the indentation index and the erythema (*p* < 0.001) of the treated scars when assessed using the Antera camera.	mild pain
Camouflage of Postburn Scarring Alopecia Using Nanofat Grafting and Follicular Unit Hair Transplantation	Evin et al. [16]	18 months following nanofat injection	the dimension of scarring alopecia (evaluated using ImageJ software (National Institutes of Health, Bethesda, MD, USA; https://imagej.nih.gov/ij/, accessed 21 May 2026), POSAS, and a 5-point Likert satisfaction scale	The average Likert cosmetic satisfaction score was 1.28 ± 0.46 before fat grafting and 4.83 ± 0.38 at 18 months after nanofat injection (*p* < 0.00001). The average POSAS scores were 6.87 ± 0.53 and 6.68 ± 0.55 before fat grafting and 2.62 ± 0.33 and 2.53 ± 0.34 after nanofat. Improvements in POSAS scores were statistically significant (*p* < 0.00001 for patients; *p* < 0.00001 for observers). Scar tissues were softer and more flexible, and their color was similar to that of the surrounding healthy skin.	none
The Investigation of the Efficacy and Safety of Stromal Vascular Fraction in the Treatment of Nanofat-Treated Acne Scar: a Randomized Blinded Controlled Clinical Trial	Behrangi et al. [24]	1 month, 3 months	scar size (1 month), dermal thickness (U/S after 3 months), acne scar grade (Goodman and Baron system graded 1 to 4)	In the case group (injected side), facial volume, area, and depth decreased significantly over time (*p* < 0.05), with epidermal, dermal, and total skin thickness showing significant improvement at 1 month (*p* < 0.05). In the control group, sonographic measures improved at 1 month (*p* < 0.05). At 3 months, dermal and total thickness remained improved in both groups. Intergroup comparisons showed that at 1 month, dermal (*p* = 0.042) and total thickness (*p* = 0.040) were greater in the case group, but no significant differences were found at 3 months.	none
Nanofat and Platelet-Rich Plasma Injections Used in a Case of Severe Acne Scars	Pons et al. [4]	1 year	Vancouver Scar Scale (VSS) and histologic analysis	The average VSS score on the treated zones was 5.67 before and 4.0 one year after. Histologic analysis of an atrophic scar before and 1 year after treatment showed a reduction of the dermal inflammatory infiltrate and an increase in the vascularization of the treated area.	none
Effect of Autologous Fat Transfer in Acute Burn Wound Management: A Randomized Controlled Study	Abouzaid et al. [1]	2 weeks, 1 month	scar texture, pigmentation, contracture formation, collagen deposition	Scar texture as well as pigmentation improved (*p* = < 0.001) in group A (nanofat dressed wounds) compared to group B (conventional serial dressings). After one month, the area percentage of collagen deposition showed a significant increase compared to day 14 in group A.	none
Application of Nanofat Grafting to Rescue a Severe Ischaemic Hand with Thromboangiitis Obliterans	Benjamin Ng et al. [14]	2 months	computed tomography angiography	Computed tomography angiography revealed a radial artery patency. The patient’s wrist function was preserved with uneventful wound healing (treatment success).	none
Autologous Nanofat Injection in Treatment of scars: A Clinicohistopathological Study	Rageh et al. [9]	6 months	VSS and histopathologic assessment (pigmentation, vascularization, elastic fiber thickness, collagen thickness and epidermal thickness) using image analysis system	Pliability (*p* = 0.006), height (*p* < 0.001) and total VSS (*p* < 0.001) scores significantly improved after treatment. All histopathological measures were significantly improved (all *p* < 0.001).	13.3% had bruising, 16.7% had edema, 10% had erythema, 1 patient (3.3%) had hyperpigmentation
Adipose Tissue Versus Stem Cell-Derived Small Extracellular Vesicles to Enhance the Healing of Acute Burns	R. Ivari and Mahdipour [8]	days 7, 14, 28	percentages of wound closure, amount of collagen and elastin in the granulation tissue, number of CD34+ vessels	NF-treated wounds showed earlier and greater granulation tissue formation, increased epithelial thickness from day 7 onward (*p* < 0.0001), and enhanced collagen deposition from day 14 to 28 compared with controls and sEV-treated wounds (*p* = 0.003). Additionally, NF strongly stimulated neoangiogenesis, with CD34^+^ vessel density in granulation tissue approximately three times higher than controls (*p* = 0.002).	none
Upper Lip Reconstruction Utilizing a Two-Stage Approach with Nanofat Grafting After Hemangioma Treatment	Qi et al. [25]	1–8 years	appearance, symmetry, and smoothness of upper lips with deformities before and after surgery	The postoperative outcomes of 9 patients who underwent fat grafting demonstrated improved lip symmetry, contour, and smoothness.	none
Nanofat Injection for the Treatment of Depressed Facial Scars	Huang et al. [12]	3 months	patient assessment FACE-Q scale (postoperative satisfaction with appearance, social function, the decision to undergo therapy and the outcome of the therapy) and surgeon scored pre-/post-photos based on “healing” (1 least healed to 4 most healed)	Compared with patients who had finished the treatment less than one year prior, patients who had finished the treatment more than one year prior showed higher satisfaction with their decision to undergo this therapy (*p* = 0.013). Patients who had finished the therapy less than one year prior showed higher satisfaction with social function than those who had finished the therapy more than one year prior (*p* = 0.046). No significant difference was found in satisfaction with the results or with appearance (*p* > 0.05). The photo comparisons revealed complete healing in 30% of cases, obvious improvement in 41%, “effective” outcomes in 20%, and no change in 9%.	Temporary erythema, lasting 2 to 3 weeks in 93% of the patients. One patient (2.3%) complained of pigmentation that developed from long-term hyperemia. Two patients (4.5%) experienced blistering
Immediate Fat and Nanofat-Enriched Fat Grafting in Breast Reduction for Scar Management	Kemaloğlu et al. [17]	6 months	Vancouver Scar Scale and patient visual analogue scores	VSS scores in pliability, pigmentation, and vascularity were significantly lower in the traditional fat graft and nanofat-enriched fat graft groups (*p* < 0.002). When comparing fat and nanofat groups pigmentation was significantly lower in nanofat group (*p* = 0.005). VAS scores were significantly lower in fat and nanofat groups (*p* = 0.001) compared to control (no treatment).	none
Adding Nanofat to Fat Grafting to Treat Velar Scarring in Velopharyngeal Incompetence	Cantarella et al. [26]	3 months	VP closure gap, perceptual speech evaluation (speech intelligibility, hypernasality, nasal air escape)	At 3 months, the patient had no more perceivable nasality or nasal turbulence during speech. At 6 months, nasoendoscopy showed complete closure of the VP sphincter and perceptual evaluation of the patient’s speech demonstrated normal values for intelligibility, hypernasality and nasal air escape.	none
Unfiltered Nanofat Injections Rejuvenate Postburn Scars of Face	Jan et al. [15]	6 months	POSAS and quantitative analysis of pigmentation levels (ImageJ)	There was a difference on POSAS between scar pliability, pigmentation, as well as overall scar score (*p* all < 0.0001). The decrease in scar pigmentation post-therapy recorded on ImageJ was not statistically significant (*p* = 0.076).	mild edema (62.5%)
Nanofat Grafting for Scar Treatment and Skin Quality Improvement	Uyulmaz et al. [27]	7 days, 3 weeks, and 3 months	patient satisfaction assessment (yes/no) and pre- and post-treatment photographs rated by physicians (good/satisfactory/no change)	Reviewers classified the results in the majority of scars post-treatment as good (74%), satisfactory (18%), and unchanged (8%). Forty-eight of our 52 patients (92%) were highly satisfied with their results.	none
LaMiNa: A Creative Synergistic Approach to Facial Rejuvenation	Claytor et al. [28]	1 day, 2 weeks, 3 months, and 6 months	pain scores, photographic comparison in fine lines and rhytids, histologic analysis	All patients verbally reported no pain (Numerical Rating System 0–10) following procedure and had rapid recovery within an average of 5 days. Pathology results demonstrated that CO_2_ and microneedling had persistent epidermal disruption and perineural inflammation at 4 days, while the introduction of autologous nanofat at the time of CO_2_ and microneedling resulted in full recovery of epidermis and resolution of perineural inflammation. Clinical observations revealed improvement in fine lines and rhytids.	none
Use of Condensed Nanofat Combined with Fat Grafts to Treat Atrophic Scars	Gu et al. [29]	6 months	POSAS, Fontana–Masson IHC staining of melanin, Verhoeff–Van Gieson IHC staining of elastic fibers, changes in sebaceous glands and sweat glands postoperatively by IHC staining using CK14 and CK19	The patients’ mean scar assessment scores were significantly decreased postoperatively in the final examination for color (*p* < 0.001); stiffness (*p* < 0.001); thickness (*p* = 0.001); and irregularity (*p* = 0.003); and the observers’ scores were also significantly decreased for pigmentation (*p* = 0.004); thickness (*p* = 0.03); relief (*p* = 0.003); and pliability (*p* < 0.001). In the final follow-up examinations, a significantly improved overall POSAS score was found among both patients (*p* < 0.001), and observers (*p* = 0.001). Enhancement of Fontana–Masson staining of melanin in the basal cell layer was observed postoperatively, and a significant postoperative change was detected for the mean values of average optical density from the preoperative measurement (*p* = 0.01). The sebaceous glands and sweat glands not found in the preoperative images were seen postoperatively by IHC staining with CK14 and CK19.	none

**Table 3:** Study Protocols.

Title	Author	Treatment Indications	Study Protocol
Role of Autologous Nanofat Grafting in the Treatment of Post Acne and Post Burn Scarring of the Face	Khan et al. [10]	Treatment of post-acne and post-burn scars	3 nanofat grafting sessions in the span of 4–6 weeks.
Evaluating the Efficacy of facial scar Treatment Techniques using Nanofat Grafting: A Case Series	Alnemr et al. [22]	Treatment of facial scars	One treatment of nanofat or nanofat with PRF.
Effectiveness of Acupotomy Subcision and Nanofat Grafting in Depressed Scars Treatment	Qu et al. [23]	Treatment of depressed facial scars	Acupotomy subcutaneous scrape, followed by Nanofat injection; three treatments, spaced out every other month.
Is Nanofat the Long-Awaited treatment for hypertensive ischemic leg ulcers?	Moris et al. [2]	Treatment of hypertensive ischemic leg ulcer (HYTILU)	Patients underwent liposuction of the abdomen with followed by intradermal injection of the nanofat at the recipient site.
Can a Combination of Nanofat and Freeze-Dried Human Amniotic Membrane Enhance full-thickness Wound Healing? An animal study using rabbit models	Hidayati et al. [18]	Treatment of full-thickness wounds in a rabbit model	Experimental group (received a combination of nanofat and FDHAM) and the control group (received FDHAM alone). Each group was subdivided to evaluate effects on days 3 and 7 post-injury.
Regenerative Nanofat Membrane Development Process	Fakih-Gomez et al. [13]	Treatment of variety of wounds (diabetic foot ulcers, cleft palate surgeries, facial dermabrasion, skin necrosis, revision rhinoplasties, and post-cosmetic surgery complications.)	Nanofat was combined with PRF to create a nanofat-PRF membrane, which was applied to the wound. Each fat membrane was tailored to match the defect dimensions.
Efficacy and Safety of Autologous Nanofat Injection in the Treatment of Postburn Scars Using Optical Skin Imaging Analysis	Rageh et al. [11]	Treatment of burn scars	One session of nanofat injection at the targeted study sites and final evaluation 4 months later.
Camouflage of Postburn Scarring Alopecia Using Nanofat Grafting and Follicular Unit Hair Transplantation	Evin et al. [16]	Treatment of post-burn scarring alopecia	Single-stage autologous nanofat injection under local anesthesia on an outpatient basis, 6 months before hair transplantation.
The investigation of the efficacy and safety of stromal vascular fraction in the treatment of nanofat-treated acne scar: a randomized blinded controlled clinical trial	Behrangi et al. [24]	Treatment of acne scars	Spilt-face study: nanofat administered subcutaneously on one side of the face (control group) and combination of nanofat and SVF was administered intradermally on the opposite side (intervention group).
Nanofat and Platelet-Rich Plasma injections used in a case of severe acne scars	Pons et al. [4]	Treatment of nodulocystic acne	Platelet-rich plasma (PRP) and nanofat mixture was injected into the pathological dermis in order to treat and fill severe acne scars.
Effect of autologous fat transfer in acute burn wound management: A randomized controlled study	Abouzaid et al. [1]	Treatment of burn wounds	Group A consisted of 50 patients treated with a single injection of autologous fat grafting, after which the wounds were dressed with nanofat, whereas group B consisted of 50 patients treated conventionally with serial dressing and topical agents.
Application of nanofat grafting to rescue a severe ischemic hand with thromboangiitis obliterans	Benjamin Ng et al. [14]	Treatment of man with cyanosis in both hands and the right foot, accompanied by severe pain	Nanofat was dispersed throughout his left forearm, from the proximal to the distal end, along both the radial and ulnar sides through intramuscular injection.
Autologous nanofat injection in treatment of scars: A clinicohistopathological study	Rageh et al. [9]	Treatment of different scar types	Patients underwent one session of Nanofat injection at the wound site until yellowish discoloration of the skin showed up.
Adipose Tissue Versus Stem Cell-Derived Small Extracellular Vesicles to Enhance the Healing of Acute Burns	R. Ivari, Mahdipour [8]	Treatment of full-thickness third-degree scald burn model in mice	Adipose tissue in the form of nanofat (NF) and MenSC-sEVs were injected subcutaneously at the burn site.
Upper Lip Reconstruction Utilizing a Two-Stage Approach with Nanofat Grafting After Hemangioma Treatment	Qi et al. [25]	Treatment of lip deformities	15 patients treated with nanofat grafting alone and 9 nanofat grafting with flap transfer. Nanofat was injected through a pinhole located at the ipsilateral corner of the mouth (most required 2–3 injections).
Nanofat Injection for the Treatment of Depressed Facial Scars	Huang et al. [12]	Treatment of depressed facial scars	The deep layer of the scar was injected first with nanofat, followed by the shallow layer (one injection).
Immediate Fat and Nanofat-Enriched Fat Grafting in Breast Reduction for Scar Management	Kemaloğlu et al. [17]	Treatment of post-surgical scars	Patients underwent breast reduction, and scars were treated immediately following surgery. Three groups: control (no treatment), fat graft injected, fat graft enriched with nanofat injected.
Adding Nanofat to Fat Grafting to Treat Velar Scarring in Velopharyngeal Incompetence	Cantarella et al. [26]	Treatment of velar scarring in velopharyngeal incompetence	Patient underwent concurrent microfat and nanofat injections in the velum, especially along the midline scar, and microfat injections in the posterior and lateral pharyngeal walls.
Unfiltered Nanofat Injections Rejuvenate Postburn Scars of Face	Jan et al. [15]	Treatment of post-burn facial scars	Fat was harvested from abdomen or flank then injected in a fanwise pattern to the scar area until the skin blanched or displayed a yellowish discoloration.
Nanofat Grafting for Scar Treatment and Skin Quality Improvement	Uyulmaz et al. [27]	Treatment of scars, wrinkles, and skin discolorations	Nanofat was applied with many small intradermal injections over the entire affected area (usually once but twice in rigid scars).
LaMiNa: A Creative Synergistic Approach to Facial Rejuvenation	Claytor et al. [28]	Improvement of fine lines and rhytids in facial skin	Patients underwent facial treatment with CO_2_ lasering, followed by microneedling and application of autologous nanofat (LaMiNa).
Use of Condensed Nanofat Combined with Fat Grafts to Treat Atrophic Scars	Gu et al. [29]	Treatment of atrophic facial scars (surgical sutures (n = 12), burns (n = 8), trauma (n = 3), and acne (n = 2)	Each scar was injected subdermally with condensed nanofat combined with fat grafts.

## Data Availability

No new data were created or analyzed in this study.

## Supplementary Materials

The following supporting information can be downloaded at: https://www.mdpi.com/article/10.3390/biomedicines14061215/s1, Table S1: Risk of Bias Assessment Table. Risk of bias was assessed based on study design using criteria adapted from the Newcastle-Ottawa Scale (NOS) for observational studies, the Cochrane Risk of Bias framework for randomized studies, and JBI critical appraisal principles for case reports/series. Studies were categorized as low, moderate, or high risk of bias based on sample size, randomization, controls, blinding, follow-up adequacy, outcome assessment, and reporting transparency; PRISMA 2020 Checklist. Reference [36] is cited in the Supplementary Material.
