# Supplementary material for: Autologous Nanofat Indications in Wound Healing: A Systematic Review
Source: Biomedicines. 2026 May 28;14(6):1215. doi: 10.3390/biomedicines14061215 (PMC13295952; doi:10.3390/biomedicines14061215)
Supplement: Supplementary file 1 [file biomedicines-14-01215-s001.zip › Table S1 Risk Assesment Table .pdf]

Table S1: Risk of Bias Assessment Table. Risk of bias was assessed based on study design using criteria adapted from the Newcastle-Ottawa Scale (NOS) for observational studies, the Cochrane Risk of Bias framework for randomized studies, and JBI critical appraisal principles for case reports/series. Studies were categorized as low, moderate, or high risk of bias based on sample size, randomization, controls, blinding, follow-up adequacy, outcome assessment, and reporting transparency.

Study	Study Design	Main Limitations	Overall Risk of Bias
Khan et al., 2024	Case Series	No control group, subjective outcomes, small sample size	High
Alnemr et al.	Case Series	Small sample(n=4), no comparator, subjective cosmetic assessment	High
Qu et al., 2025	Retrospective Analysis	Retrospective design, single-center study, limited blinding	Moderate
Moris et al., 2025	Retrospective Pilot Study	Pilot design, no control group, small cohort	High
Hidayati et al., 2024	Animal experimental study	Animal model limits external validity, short follow-up	Moderate
Fakih-Gomez et al., 2025	Retrospective Analysis	Heterogeneous wounds and procedures, no standardized controls	High
Rageh et al., 2025	Prospective trial	No randomization or control group	Moderate
Evin et al., 2024	Patient Series	Small sample size, no comparator group	High
Behrangi et al.,	Randomized single-blinded clinical trial	Very small sample size (n=7), short follow-up	Moderate
Pons et al., 2022	Case report	Single patient, no control, highly limited generalizability	High
Abouzaid et al., 2021	Randomized control trial	Single-center design, open-label methodology	Low

Benjamin Ng et al., 2021	Case report	Single patient, adjunctive procedures confound outcomes	High
Rageh et al., 2021	Prospective trial	No control group, subjective scar assessment	Moderate
Rohani et al., 2021	Randomized animal trial	Animal model limits translation to humans	Moderate
Qi et al., 2021	Retrospective Study	Retrospective design, no comparison group	Moderate
Huang et al., 2021	Retrospective study	Subjective outcomes, retrospective methodology	High
Kemaloglu et al., 2021	Prospective single-center study	Lack of blinding, limited sample size	Moderate
Cantarella et al., 2020	Case report	Single patient, adjunctive fat grafting confounds findings	High
Jan et al., 2018	Prospective Study	No control group, subjective outcomes	Moderate
Uyulmaz et al., 2018	Retrospective study	Retrospective design, heterogeneous scars	High
Claytor et al., 2023	Prospective Study	Combined therapies confound isolated Nanofat effect	Moderate
Gu et al., 2018	Prospective case series	Small sample size, no control group	High
